# The value of genomic testing in severe childhood speech disorders

**DOI:** 10.1038/s41431-024-01534-w

**Published:** 2024-02-02

**Authors:** Yan Meng, Stephanie Best, David J. Amor, Ruth Braden, Angela T. Morgan, Ilias Goranitis

**Affiliations:** 1 The University of Melbourne, Parkville, VIC Australia; 2 Australian Genomics Health Alliance, Melbourne, VIC Australia; 3 Peter MacCallum Cancer Center, Parkville, VIC Australia; 4 Victorian Comprehensive Cancer Center, Parkville, VIC Australia; 5 Murdoch Children’s Research Institute, Parkville, VIC Australia; 6 Royal Children’s Hospital, Parkville, VIC Australia

**Keywords:** Health care economics, Genomics

## Abstract

With increasing gene discoveries for severe speech disorders, genomic testing can alter the diagnostic and clinical paradigms, enabling better life outcomes for children and their families. However, evidence on the value of the outcomes generated is lacking, impeding optimal translation into health care. This study aims to estimate the value and uptake of genomic testing for severe childhood speech disorders. A discrete choice experiment was undertaken to elicit preferences for genomic testing from the perspective of the Australian public (*n* = 951) and parents of children experiencing severe speech disorder *(n* = 56). Choice attributes associated with genomic testing were identified through focus groups. A Bayesian D-efficient design was used to develop choice scenarios and choice data were analyzed using a panel error component mixed logit model and a latent class model. Statistically significant preferences were identified across all seven attributes. The mean monetary value of the benefits of genomic testing relative to standard diagnostic care in Australia was estimated at AU$7489 (US$5021) and AU$4452 (US$2985) from the perspectives of the Australian public and families with lived experience of severe speech disorders, with a corresponding test uptake of 94.2% and 99.6%. To ensure fair prioritization of genomics, decision-makers need to consider the wide range of risks and benefits associated with genomic information.

## Introduction

Childhood apraxia of speech (CAS) is a complex neurodevelopmental disorder affecting 1 to 2 per 1000 children, in which the ability to plan and sequence speech movements is impaired, thereby decreasing the precision, consistency and intelligibility of speech [1, 2]. Children with CAS have severely impaired speech development and need explicit teaching and practice of every new sound and word, with hours of weekly speech therapy, often until adolescence [3]. Even with years of intensive therapy, CAS can be life-long, with deleterious impacts on psychosocial, literacy, educational and employment outcomes [4]. However, current speech therapy is often ineffective because it uses trial and error approaches targeting symptoms rather than the cause. There is little consensus on the etiology of severe speech disorders, preventing the use of targeted therapies [5]. Families search for explanatory causes in a long diagnostic journey, attending to multiple GP, pediatrician, neurology, and other specialist appointments involving various investigations with significant cost and psychological stress without explanatory results [6]. Arguably, even greater costs are seen in speech therapy sessions and missed school and work for attending appointments [7].

One of the challenges of managing CAS has been a lack of understanding of the condition’s etiology. Recently, there have been remarkable gene discoveries in this field, showing a substantial contribution of genetic risk from single gene variants [8–12]. Research suggests that genomic testing produces a diagnostic yield in as many as one in three children [13]. A genomic diagnosis can enable a change in the clinical paradigm of speech disorders, moving from the current ‘watch and wait’ surveillance or trial and error symptom-based therapies for CAS towards precision medicine, which can optimize life outcomes for children [14].

Genomic testing can provide significant personal utility to patients and their families, and providing this information to decision makers is key in ensuring that genomic technologies and people experiencing conditions with underlying genetic cause are not disadvantaged in resource allocation decisions [15–19]. While quantitative evidence for the value of genomic testing to patients and the public has started to emerge [17–20], and to inform health economic evaluations [21, 22], no evidence yet exists in the context of severe speech disorders. To address this gap, this study aims to elicit preferences and value for genomic testing in severe childhood speech disorders from the perspectives of the Australian public and parents of children experiencing severe speech disorders. Our findings demonstrate the relative priority of the different characteristics of genomic testing that matter to people and enable an estimation of the uptake and value of genomic testing for severe speech conditions. The findings will facilitate a cost-benefit analysis to inform the translation of genomic testing into the Australian health care system.

## Materials and methods

### Study design and participants

We conducted a discrete choice experiment (DCE) to elicit preferences for different attributes associated with genomic testing in the context of severe speech disorders. Following best practice recommendations, focus groups were conducted with parents of children experiencing severe speech disorders to identify attributes that were deemed important for the genomic testing decision-making process [23]. Led by an experienced facilitator (SB), deliberative focus groups to identify and refine attributes were guided by existing genomic testing literature, expert opinion and focus groups, involving qualitative discussions and quantitative rating [24]. The final set of attributes were developed in consultation with the broader research team (YM, IG, AM, DA, SB) to ensure clinical face validity, as shown in Table 1.

**Table 1:** Attributes and levels included in the discrete choice experiment (DCE).

No.	Attributes	Levels
1	Number of children who receive genetic diagnosis	20 out of 100
		30 out of 100
		40 out of 100
		50 out of 100
2	Knowledge about the child’s future health and development (prognosis)	No knowledge
		Some knowledge
		A lot of knowledge
3	Chance of improving the process of the child’s medical care	20%
		30%
		40%
		50%
4	Time between now and when your child does the test	1 month
		3 months
		6 months
5	Cost of testing to you	A$500
		A$1,500
		A$3,000
		A$4,500
6	Allowing access to educational support services	Yes
		No
7	Enabling access to relevant genetic-based family support groups	Yes
		No

A DCE survey was administered to a representative sample of the Australian adult population (public survey) and parents of children with a severe speech disorder (parent survey). The content of the surveys included demographic questions, text about genomic testing in severe speech disorders, a detailed description of the attributes and levels, as well as an example choice task to facilitate participants’ understanding of the process in evaluating the choice scenarios. The distinction between the two surveys was that additional information was provided in the public survey about severe speech disorders, including a video of a child with CAS, due to the public’s relative lack of familiarity with the condition of interest. To minimize hypothetical bias caused by respondents failing to fully consider the consequences of decisions, we inserted a cheap talk script following Cummings and Taylor [25].

Following Weber [26], the survey was coded and built in Stata and HTML by the research team on the Qualtrics platform and was largely automated to present the choice tasks. The public survey was piloted for comments and feedback with 115 members of the Australian public recruited by Qualtrics. The parent survey was piloted with our 14 original parent focus group members. During the pilot test, we asked respondents about the design, readability and level of the difficulty of the survey. At the end of the survey, we provided a free-text option for any further feedback they would like to share. Wordings were refined based on the pilot feedback to improve the clarity. The final version of the survey was approved by all members of the research team and focus group members. The public survey is provided in the online Supplementary Material.

### Experimental design

**Fig. 1:**
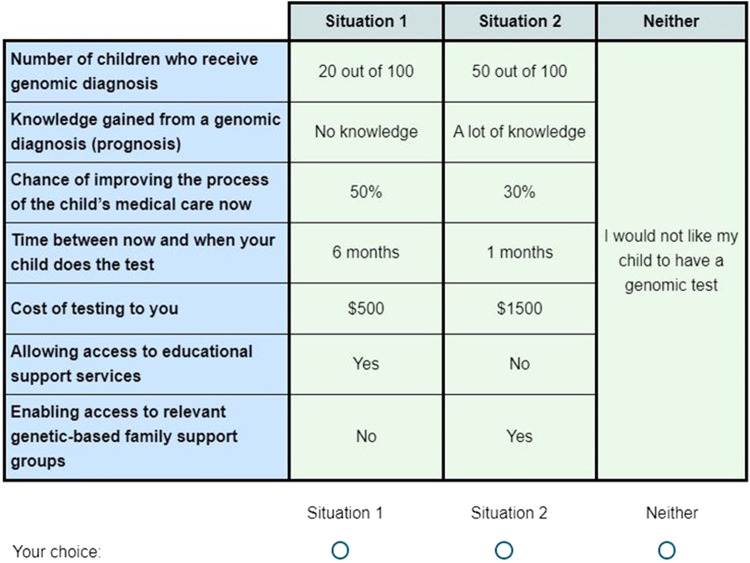
An example of the choice tasks.

We adopted a Bayesian D-efficient design using Ngene to accommodate the uncertainties associated with parameter values to ensure the parameters were robust against prior misspecification. Priors were informed by the pilot results and published evidence [27]. The choice tasks were administered in 4 blocks, wherein each participant was randomized to 1 block consisting of 12 choice tasks. Blocking was performed to reduce task effort for the respondents and was implemented using the minimum correlation principle. Each choice task asked participants to indicate the situation under which they would choose to undergo genomic testing. Participants could choose among three options (Situation 1, Situation 2 or Neither). The “Neither” option displayed “I would not like my child to have a genomic test”. An example of the choice tasks is shown in Fig. 1.

For the public survey, participants over the age of 18 were recruited from existing nationwide panels maintained by the survey vendor across Australia. Age, gender and household income quotas were applied to ensure the representativeness of the sample. Target sample sizes were determined based on the S-efficiency measure, which is the minimum sample size required to achieve significant results at 5% level of significance, conditioning on the correct specification of the priors [28]. Respondents in the parent survey were recruited by the research team via email from the current Genetics in Speech project or the Murdoch Children’s Research Institute and the Royal Children’s Hospital in Melbourne, Australia.

### Statistical analyses

Descriptive statistics were used to examine the demographic and socioeconomic characteristics of the recruitment samples. Choice data were analyzed using a panel random parameter error component model in Nlogit 6. Random parameters were used to account for unobserved heterogeneity of preferences of individual respondents and error component accommodated for choice correlation that was related to the alternatives [29]. In the model specification, cost and time were assumed continuous variables, and their parameters followed constrained triangular distributions. All other attribute levels were dummy coded, and their associated parameters were assumed to be normally distributed. Random parameters were estimated using 500 Halton draws. Attribute importance were calculated and normalized to reflect the absolute change in utility associated with an attribute relative to the aggregated utility change [30]. Marginal willingness-to-pay for each attribute was calculated based on conditional (individual-level) estimates. Due to the uncertainty regarding which alternative will be chosen in a DCE, the uptake and total willingness-to-pay for genomic testing were derived based on compensating variation formula [31]. Values were reported in Australian dollars.

## Results

### Focus group results

Five focus groups were held between May and June 2021. The first three focus groups centered on attribute identification and the last two concentrated on attribute refinement. We invited 31 parents, who participated the Genetics of Speech Disorders Study at the Murdoch Children’s Research Institute or the Speech Genetics clinic at the Royal Children’s Hospital, and had consented to be contacted about future research studies related to child speech disorders. Parents were contacted by phone and invited to attend one or two of five focus group sessions (one based on attribute identification and one on attribute refinement), based on their availability. Of the 31 invited to participate, 18 agreed to participate and provided written consent, and 14 attended one of the first three sessions about attribute identification. Six parents attended one of the last two sessions focusing on attribute refinement. From the 14 unique participants, 5 had a variant identified, 2 had undergone testing but no result as yet, and 7 had yet to complete whole genome sequencing or whole exome sequencing.

A range of health (e.g., identifying a child’s prognosis) and non-health (e.g., altruistic contribution to research) characteristics that may influence the decision of genomic testing in complex speech conditions were identified and refined through focus groups. Some de-identified exemplar quotes were generated with pseudonyms at the end of each quote.

1. the number of children who receive a genetic diagnosis, *If in future, someone who is unsure whether their child has this is able to go and get a genetic test and go, yep, you’ve got it, let’s start moving and progressing, and you don’t have to go through all that effort of trying to find the right person to run the right tests, and it’s quite subjective. Whereas if there is a genetic link and they can find that with this research, that they can get started quicker*. FG2 Sharon
2. knowledge gained from a genomic diagnosis (e.g., such as prognosis), *I think that if the diagnosis, the genomic testing, can lead to a diagnosis that has treatment outcomes, the sooner you know the better*. FG2 Rita
3. chance of improving the process of the child’s medical care now, *Suddenly the health professionals and the allied professionals basically said, oh yeah, okay, she really has got something, and were then willing to work with me*. FG1 Lorna
4. time between now and when your child undergoes the genetic testing, *it’s decreasing the time it takes to access the support*. FG5 Kelly
5. cost of testing to the individual, *I found out the cost of genomic testing and that really threw me off*. FG3 Alison
6. allowing access to educational support services, *You have to write the diagnosis on your application form for school, and that leads to supports that he’ll need in class. So that’s a huge reason as to why you’d need a diagnosis*. FG2 Fiona
7. enabling access to relevant genetic-based family support groups. *I think a positive outcome of doing genetic testing is then to be able to connect with other families that have potentially similar experiences*. FG1 Susan

### Survey respondent characteristics

In total, we had 951 respondents (cooperation rate = 69%) from the Australian public. For the parent survey, 128 parents were invited to take part and 56 completed the survey (overall response rate of 44%). As shown in Supplementary Table S1, respondents in the public sample had an average age of 41 years (range = 18 to 88, SD = 18.4) with 53% being female, 55% married or in a de facto relationship, and 37% having a university degree. Around half of the respondents had a household income over AU$91,000. Of the 951 respondents, 220 (23%) had prior knowledge or experience about severe speech disorders like apraxia or dysarthria, and 215 (23%) had prior knowledge or experience of genomic testing. For the sample of parents of children with lived experiences of severe speech disorders, mean age was 41 (range = 31 to 62, SD = 7.2). More than half of the respondents (54%) had private health insurance and 91% of them had access to the National Disability Insurance Scheme Fund. Additional information about the two samples was provided in Supplementary Table S1.

The regression results from the public survey are presented in Table 2. All coefficients were significant at 1% level of significance. Overall, respondents demonstrated strong preferences for genomic testing when the number of children who receive genomic diagnosis increased, when more knowledge could be gained from the genomic diagnosis, and when there was a higher chance to improve the process of the child’s medical care now. Respondents also preferred genomic testing when it enabled educational support services or relevant genetic-based family support groups. As, expected, there was a disutility associated with time until the child does the test and cost, as shorter waiting time and lower cost are generally preferred. The standard deviation of all parameters from the random parameter model were all statistically significant, indicating preference heterogeneity among respondents. As shown in Table 3, despite the small sample size of the parent survey, all parameters were statistically significant except for enabling access to relevant genetic-based family support groups.

**Table 2:** Marginal utilities and willingness to pay (WTP) of the public.

	Mean^a^	Standard deviation^b^	importance score, %	Marginal WTP (AU$)^c^
Number of children who receive genetic diagnosis	0.01061***	0.02124***	9	45
Knowledge gained from a genomic diagnosis (some knowledge)	0.37490***	0.24051**	11	1478
Knowledge gained from a genomic diagnosis (a lot of knowledge)	0.79262***	0.84583***		3092
Chance of improving the process of the child’s medical care now	0.02224***	0.03137***	18	90
Time between now and when your child does the test	−0.04976***	0.04976***	7	−201
Cost of testing to you	−0.00038***	0.00038***	41	
Allowing access to educational support services	0.34912***	0.49582***	9	1140
Enabling access to relevant genetic-based family support groups	0.15591***	0.32868***	4	520
constant	1.45218***	0.17476^d^		
sigma^e^	4.39131***			
Log likelihood	−8709			
McFadden Pseudo R-squared	0.3			

***p < 0.01; **p < 0.05; *p < 0.1

^a^Marginal utilities indicate the marginal effect of each attribute (or attribute level) on the utility for genomic testing. Positive (or negative) mean estimates indicate, on average, a positive (or negative) effect on utility.

^b^Standard deviation estimates describe the heterogeneity of preferences among study participants.

^c^Marginal WTP estimates represent the marginal rate of substitution between the corresponding attribute and the cost attribute.

^d^Constant is specified as a fixed parameter so this value here is the standard error of the parameter estimate.

^e^Random parameter associated with the genomic testing alternatives.

**Table 3:** Marginal utilities and willingness to pay (WTP) of parents of children with lived experiences of severe speech disorders.

	Mean^a^	Standard deviation^b^	importance score, %	Marginal WTP (AU$)^c^
Number of children who receive genetic diagnosis	0.02939*	0.03664*	8	49
Knowledge gained from a genomic diagnosis (some knowledge)	1.60958***	0.29275	11	2352
Knowledge gained from a genomic diagnosis (a lot of knowledge)	2.84025***	1.12270*		4207
Chance of improving the process of the child’s medical care now	0.05836***	0.04728***	15	93
Time between now and when your child does the test	−0.11380**	0.11380**	5	−169
Cost of testing to you	−0.00124***	0.00124***	43	
Allowing access to educational support services	1.65854***	1.39929***	14	3094
Enabling access to relevant genetic-based family support groups	0.45026	0.6997	4	658
Constant	1.02101	1.04987^d^		
Sigma^e^	4.89507***			
Log likelihood	−296			
McFadden Pseudo R-squared	0.4			

***p < 0.01; **p < 0.05; *p < 0.1

^a^Marginal utilities indicate the marginal effect of each attribute (or attribute level) on the utility for genomic testing. Positive (or negative) mean estimates indicate, on average, a positive (or negative) effect on utility.

^b^Standard deviation estimates describe the heterogeneity of preferences among study participants.

^c^Marginal WTP estimates represent the marginal rate of substitution between the corresponding attribute and the cost attribute.

^d^Constant is specified as a fixed parameter so this value here is the standard error of the parameter estimate.

^e^Random parameter associated with the genomic testing alternatives.

Tables 2 and 3 also present additional information about the relative importance of each attribute. Based on the public preference, the chance of improving the process of the child’s medical care now was of particular importance apart from cost, followed by knowledge gained from a genomic diagnosis, number of children who receive genetics diagnosis and allowing access to educational support services. We observed similar patterns of attribute importance in the sample of parents with lived experience of severe speech disorders, except that allowing access to educational support services was the third most important attribute for this group when making choices.

The marginal willingness-to-pay of different attributes of genomic testing are presented in Tables 2 and 3. On average, the public was willing to pay: AU$45 (US$30) for every additional child in a hundred receiving a genetic diagnosis; AU$1478 (US$991) to gain some knowledge from a genetic diagnosis and AU$3092 (US$2073) to gain a lot of knowledge compared with no knowledge; AU$90 (US$60) for every percentage point increase in the chance of improving the process of the child’s medical care now; AU$201 (US$135) to take the test one month sooner; AU$1140 (US$764) to access educational support services and AU$520 (US$349) to access relevant genetic-based family support groups. Parents of children with lived experience of severe speech disorders placed a significantly higher value on educational support services (AU$3094 [US$2075] vs AU$1140 [US$764]). Parents of children with speech conditions here also greatly valued the knowledge gained from a genomic diagnosis. They were willing to pay AU$2352 (US$1577) to gain some knowledge, which is 1.6 times higher than the willingness-to-pay from the public. To gain a lot of knowledge, this value went up to AU$4207 (US$2821), 1.4 times higher than that of the public. Similar values between the two samples were observed for other attributes.

Uptake and willingness-to-pay for genomic testing of severe speech disorders based on the current knowledge and evidence of the diagnostic utility, clinical utility, as well as other elements associated with the test such as time to test and whether it enables access to educational support services and family support groups are outlined in Supplementary Table S2. Cost was assumed to be zero (i.e., no out-of-pocket cost for accessing the test) in the calculation to facilitate future cost-benefit evaluations, because genomic testing was treated as part of a publicly-funded healthcare system. On average, the willingness-to-pay for genomic testing of the public is AU$7489 (US$5021), with an uptake of 94.2%. For parents of children with severe speech disorders, 99.6% were predicted to choose genomic testing over standard care with an average willingness-to-pay of AU$4452 (US$2985).

Preference heterogeneity among the Australian public can be further demonstrated based on the results of the latent class panel model shown in Table 4, through which we identify 3 distinct classes. Individuals in classes 1 and 2 constitute the majority of the sample (60% and 26%, respectively) and there is a 14% chance that a respondent falls into class 3. All attributes are statistically significant, meaning respondents were willing to trade off different aspects of genomic testing against each other when making their choices. Respondents in class 1 demonstrated a stronger preference for testing, reflected by the positive and significant genomic testing constant, whereas respondents in class 2 were more price sensitive and especially valued access to educational support services. Conversely, respondents in class 3 did not value genomic testing, and would not consider genomic testing regardless of the changes in attribute levels.

**Table 4:** Marginal utilities based on the latent class models.

	Class 1		Class 2		Class 3	
Attribute	Coefficient	Standard error	Coefficient	Standard error	Coefficient	Standard error
Number of children who receive genomic diagnosis	0.0087***	0.0014	0.0079***	0.0025	−0.0192	0.0233
Knowledge gained from a genomic diagnosis (a lot of knowledge)	0.3064***	0.0399	0.4810***	0.0808	0.5755	0.6991
Knowledge gained from a genomic diagnosis (some knowledge)	0.6679***	0.0411	0.7109***	0.0809	0.1119	0.7994
Chance of improving the process of the child’s medical care now	0.0194***	0.0016	0.0135***	0.0027	0.0120	0.0246
Time between now and when your child does the test	−0.0318***	0.0075	−0.0342**	0.0138	−0.1542	0.1390
Cost of testing to you	−0.0002***	0.00002	−0.0005***	0.00003	−0.0003	0.0002
Allowing access to educational support services	0.2933***	0.0291	0.4213***	0.0604	0.4350	0.5299
Enabling access to relevant genetic-based family support groups	0.1390***	0.0298	0.1687***	0.0612	0.0944	0.5957
Genomic testing constant	2.1355***	0.1515	−0.4194**	0.1802	−4.3946***	1.5026
Class probabilities	0.60***	0.0175	0.26***	0.0160	0.14***	0.0113
Pseudo R-squared	0.30					
AIC	17700					

## Discussion

This paper estimated the uptake and value of genomic testing in the context of severe speech disorders (e.g., CAS) from the perspectives of the Australian public (*n* = 951) as well as families with lived experiences of severe speech disorders (*n* = 56), using a random parameter error component model. Both the public and parents prefer genomic testing when there is an increased diagnostic yield, higher clinical utility, faster access to tests, lower cost and when there is educational support services or relevant genetic-based family support groups. We estimated that on average, the Australian general public is willing to pay AU$7489 (US$5021), with a predicted genomic testing uptake of 94.2%, while families with lived experience of severe speech disorders were estimated to be willing to pay on average AU$4452 (US$2985), with an uptake of 99.6%.

A growing number of studies have demonstrated the high value that society and affected families place on genomic testing using the DCE method. Goranitis et al. [18] found that the Australian public (*n* = 820) would be willing to pay AU$5650 for genomic testing relative to standard care for complex pediatric neurological disorders. In another study [17], societal willingness-to-pay (*n* = 533) for different scenarios involving pediatric genetic conditions was estimated to range between $5470–$15,250 (US$3830–$10,675) depending on the benefits of genomic information. For critically ill infants and children, the value of genomic testing relative to standard diagnostic care ranged between AU$9810–$11,500 (US$6657–$8050) depending on the results turnaround time. Marshall et al. [32] elicited preference for diagnostic testing from parents of children with rare diseases of suspected genetic conditions in Canada (*n* = 319) and found that parents would be willing to pay CAD$6590 (US$4943) to obtain a diagnostic result.

Our overall willingness-to-pay estimate from the public is broadly consistent with previous research and reflects that respondents highly value genomic testing. For severe speech disorders (e.g., CAS), where targeted speech therapy is critical, a genomic diagnosis is useful in guiding the medical management of the child’s speech disorder or other aspects of their general health. The average willingness-to-pay of AU$4452 (US$2985) from families with lived experience is very close to the current clinical exome test (trio) price of AU$4100 if this test was not made available for free. We believe this market price might explain why the parents of the patients’ willingness-to-pay was lower than that of the public. That is, families with lived experience had all been part of a genomic testing study where they were made aware of the current market price of testing. There is a body of literature providing insights into reference prices and how they modulate willingness-to-pay [33]. Reference prices are formed by the consumers’ past experience or current purchasing environment and respondents in the parent survey may have remembered or are aware of the price in the market. The price then can be set as an anchor and significantly influence the willingness-to-pay [34]. In completing the DCE parent survey, respondents may have used the market prices as an initial anchor and adjusted their choices based on the additional information provided by other attribute levels.

While current evidence suggests that clinical utilities alone may not be sufficient to measure the value people place on genomic testing [35, 36], our findings are consistent with the existing body of literature demonstrating the need to include nonclinical outcomes to evaluate the total benefit, such as waiting time to test, level of knowledge gained about the condition, and access to peer support or advocacy groups [15, 18, 19]. Timely access to the test may shorten the period of uncertainty about the cause of the speech disorder, reducing the need for further tests and provide evidence on the appropriateness of current therapies, therefore creating better opportunities to improve the child’s speech or general health [37]. Knowledge gained from a diagnosis may be able to provide specific information about how the child’s speech disorder is likely to progress or whether it is likely to be accompanied by other health problems or learning difficulties. This knowledge can reduce parents’ uncertainty and enable future planning. In terms of peer support, although we did not find it significantly influence the preference of genomic testing from the parent perspective, other research has demonstrated that regular meetings and discussion of shared experiences in raising children with the same difficulties are known to improve the wellbeing of both children and their families [38]. Genetic-based support groups also help to advocate for children and families with the condition in terms of lobbying for support, recognition and funding. They often support research and development of therapies and knowledge around the condition [39]. Further research is warranted to explore the heterogeneity of parent preferences via a larger sample. In evaluating genomic testing, it is necessary to capture benefits such as these that go beyond health outcomes.

This study particularly identified the significance of allowing access to educational support services through genomic testing. The test results may help the child’s school and teachers better understand their speech disorder and provide tailored support to their educational needs, such as establishing and planning for the educational goals and devising appropriate adaption of the curriculum, or even enable early interventions in an educational setting, and may provide further support options for the child in special schools. Given the difficulty accessing speech pathology services, a greater focus is needed on providing educational staff with the knowledge, skills and resources to strengthen inclusion of children with severe speech disorders into mainstream education [40], and this goal is arguably better facilitated by further information around the etiology of a child’s condition. As evidenced in both the focus group discussion and the online survey feedback, parents found it important to ensure inclusive and equitable learning opportunities for all.

Furthermore, the results of the latent class analysis revealed classification of patients into three distinct groups: those who had a strong preference for genomic testing; those who preferred genomic testing especially the benefit of enabling educational support services but more cost sensitive; and those who did not value genomic testing. By considering the diverse preferences of patients with different characteristics, a more patient centered approach is needed when it comes to shared decision making.

Our inclusion of both societal and parents’ perspectives on genomic testing of severe speech disorders, specifically CAS, is an important addition to the emerging literature on preference elicitation in this field. Although our study has several strengths, there are some limitations. First, a common limitation of DCEs is that choices made under each scenario are hypothetical and may not directly replicate the choices that would be made in a real-life scenario. In the survey design, we aimed to improve external validity by inserting cheap talk scripts to ensure the comparability of hypothetical and actual choices [41]. We also included a video of a child with CAS in the public survey to increase the understanding of this condition. Second, respondents of the public survey were recruited through a market research company. This may result in self-selection or incentive biases. Respondents to the parent survey were the families of children who have severe speech disorder and who have all had genetic testing, with findings in one third of families. Their views may not be representative of parents whose children have not undergone genomic testing. Lastly, we did not control for where families are currently placed in their child’s diagnostic journey. Parents who have been searching for answers for years may place a higher value on genomic testing than those who have just begun their journey.

In conclusion, our study provides empirical evidence for the personal utility and uptake of genomic testing in the context of severe speech disorders (e.g., CAS), recognizing the relevance of a wide range of health and non-health outcomes. It demonstrates the importance of incorporating personal utility in evaluating the merits of undergoing genomic technologies. The findings will facilitate a cost-benefit analysis to inform the translation of genomic testing into the Australian health care system.

### Supplementary information

Supplementary material_tables

Supplementary material_survey

## Data Availability

The code used for the analysis is available on request, and the dataset used for estimating the willingness-to-pay for genomic testing is available upon request with the limitation that availability of individual level data is subject to consent/privacy policies from the individual clinical cohorts.
