# Supplementary material for: The value of genomic testing in severe childhood speech disorders
Source: Eur J Hum Genet. 2024 Feb 2;32(4):440–7. doi: 10.1038/s41431-024-01534-w (PMC10999408; doi:10.1038/s41431-024-01534-w)
Supplement: Supplementary file 1 — Supplementary material_tables [file 41431_2024_1534_MOESM1_ESM.docx]

**Table S1** Demographic and attitudinal characteristics

|  | Public survey (N = 951) | | Parent survey (N = 56) | |
| --- | --- | --- | --- | --- |
|  | Number | Percentage | Number | Percentage |
| **Age** | mean = 41; SD = 18.4 | | mean = 41; SD = 7.2 | |
| **Gender (Female)** | 506 | 53.2 | 55 | 98.2 |
| **Marital status** |  |  |  |  |
| Never married, separated, or widowed | 428 | 45.0 | 3 | 5.4 |
| Married or in a de facto relationship | 523 | 55.0 | 53 | 94.6 |
| **Educational status** |  |  |  |  |
| Year 11 or below | 114 | 12.0 | 1 | 1.8 |
| Year 12 or equivalent | 205 | 21.6 | 3 | 5.4 |
| Certificate | 163 | 17.1 | 14 | 25.0 |
| Diploma/advanced diploma | 120 | 12.6 | 3 | 5.4 |
| Bachelor’s degree | 248 | 26.1 | 20 | 35.7 |
| Graduate diploma/certificate | 43 | 4.5 | 1 | 1.8 |
| Post-graduate degree | 58 | 6.1 | 14 | 25.0 |
| **Annual household income** |  |  |  |  |
| Lower than $26,000 per year | 130 | 13.7 | 0 | 0 |
| $26,000 - $52,000 per year | 195 | 20.5 | 5 | 8.9 |
| $52,000 - $91,000 per year | 209 | 22.0 | 5 | 8.9 |
| $91,000 - $156,000 per year | 221 | 23.2 | 26 | 46.4 |
| $156,000 - $208,000 per year | 132 | 13.9 | 13 | 23.2 |
| Over $208,000 per year | 64 | 6.7 | 7 | 12.5 |
| **Number of children** | 1.3 | 1.5 | 2.9 | 1.1 |
| **Private health insurance (Yes)** | 535 | 56.3 | 30 | 53.6 |
| **Adult health score (0-100)** | mean = 68; SD = 20.5 | | mean = 74; SD = 16.9 | |
| **Children health score (0-100)** | mean = 84; SD = 16.4 | | mean = 85; SD = 11.4 | |
| **Knowledge of severe speech disorders (Yes)** | 220 | 23.1 | - | - |
| **Knowledge of genomic testing (Yes)** | 215 | 22.6 | - | - |
| **Access to National Disability Insurance Scheme (NDIS) fund** | - | - | 51 | 91.1 |

**Table S2** Current knowledge, uptake and willingness-to-pay (WTP) of public and patient for genomic testing

| **Attributes** | **Levels** | **Current knowledge** | **Values used for calculation of WTP** |
| --- | --- | --- | --- |
| Number of children who receive genetic diagnosis | 20 out of 100 | 30 out of 100 | 30 out of 100 |
|  | 30 out of 100 |  |  |
|  | 40 out of 100 |  |  |
|  | 50 out of 100 |  |  |
| Knowledge about the child's future health and development (prognosis) | No knowledge | 10% of people who receive a diagnosis | 3% |
|  | Some knowledge | 30% of people who receive a diagnosis | 9% |
|  | A lot of knowledge | 60% of people who receive a diagnosis | 18% |
| Chance of improving the process of the child’s medical care | 20% | 80% of people who receive a diagnosis | 24% |
|  | 30% |  |  |
|  | 40% |  |  |
|  | 50% |  |  |
| Time between now and when your child does the test | 1 month | 3 months | 3 |
|  | 3 months |  |  |
|  | 6 months |  |  |
| Cost of testing to you | A$500 | No out-of-pocket cost | 0 |
|  | A$1,500 |  |  |
|  | A$3,000 |  |  |
|  | A$4,500 |  |  |
| Allowing access to educational support services | Yes | 90% of people who receive a test | 90% |
|  | No | 10% of people who receive a test | 10% |
| Enabling access to relevant genetic-based family support groups | Yes | 90% of people who receive a test | 90% |
|  | No | 10% of people who receive a test | 10% |
|  |  |  |  |
| Mean uptake | Public: 94.2% | Patient: 99.6% |  |
| Mean WTP | Public: AU$7489 | Patient: AU$4452 |  |
