# Supplementary material for: The value of genomic testing in severe childhood speech disorders
Source: Eur J Hum Genet. 2024 Feb 2;32(4):440–7. doi: 10.1038/s41431-024-01534-w (PMC10999408; doi:10.1038/s41431-024-01534-w)
Supplement: Supplementary file 2 — Supplementary material_survey [file 41431_2024_1534_MOESM2_ESM.docx]

**Understanding preferences around genomic testing for severe speech disorders in children**

We are interested in understanding society’s views on the importance of the information and benefits that genomic testing could offer to children with severe speech disorders and their families. As a member of the Qualtrics panel, we are inviting you to do a survey about genomic testing for finding an explanatory cause for the child’s speech disorder.

Genomic tests examine our genes. Genes contain information that makes us who we are, including our eye colour and gender. Many health conditions are caused by changes in our genes. Genomic tests can screen all our genes to identify these changes and inform the prevention or treatment of a health condition.

Families who have children with severe speech disorders, can benefit from genomic testing, as it is possible that there is a genetic cause to the child’s speech disorder. Research suggests around 1 in 3 children with severe speech disorder may have a genetic cause for their condition.^[[1]](#footnote-1)^ With this survey, we want to understand your views about the importance of the different information and benefits that genomic testing may provide to children with severe speech disorders and their families.

The survey will take about 20 minutes to complete.

Your participation is voluntary. Your responses will be confidential and will not be shared with anyone outside the research team. This research has ethical approval from the Medicine and Dentistry Human Ethics Committee of the University of Melbourne. You can withdraw from this research at any time and without any consequences.

In the following page, we provide a description of the project and the nature of your participation. To enter the survey, you will need to sign a consent form. This form will allow us to use the information you provide for our research. If you have any questions, please contact A/Prof Ilias Goranitis ([ilias.goranitis@unimelb.edu.au](mailto:ilias.goranitis@unimelb.edu.au)) or Dr Yan Meng ([yan.meng2@unimelb.edu.au](mailto:yan.meng2@unimelb.edu.au)).

We appreciate your time and assistance.

**Plain Language Statement**

Centre for Health Policy/ Melbourne School of Population and Global Health

***Project: Understanding preferences around genomic testing for severe speech disorders in children***

A/Prof Ilias Goranitis; Email: [ilias.goranitis@unimelb.edu.au](mailto:ilias.goranitis@unimelb.edu.au)

Dr Yan Meng; Email: [yan.meng2@unimelb.edu.au](mailto:yan.meng2@unimelb.edu.au)

**Introduction**

We are contacting you because you are a member of the Research Now SSI panel. The following few pages will provide you with further information about the project, so that you can decide if you would like to take part in this research.

Please take the time to read this information carefully. You may ask questions about anything you don’t understand or want to know more about. Your participation is voluntary. If you don’t wish to take part, you don’t have to. If you begin participating, you can also stop at any time.

**What is this research about?**

We know the use of genomics (looking at all the genes in the body) can benefit children and families by providing a diagnosis, informing treatment, and enhancing knowledge about the condition. This may come with potential challenges, such as anxiety or uncertainty in cases where the speech disorder will not be resolved or another health condition is diagnosed. We want to understand the importance that people in the society, who may or may not have a lived experience of a genetic condition, place on the information and benefits that genomic testing can provide to children with severe speech disorders and their families. This will help us to identify how important society believes these benefits are to people who are likely to have genetic conditions and their families.

**What will I be asked to do?**

Should you agree to participate, you will be asked to complete one survey. The survey will take about 20 minutes to complete. Initially, the survey asks some background information about you. The survey then describes the information and benefits people hope to get from genomic testing in a specific clinical context. We are interested in getting public views about genomic testing on children with severe speech disorders. You will then be asked to make choices based on how important these benefits are to you.

**What are the possible benefits?**

There will be no direct benefits from your participation. However, there are great potential benefits for society. Understanding what genomic test characteristics matter to people will allow the best implementation of genomics in the Australian health system and beyond. Standard incentives for survey participation will be provided per RNSSI protocols.

**What are the possible risks?**

We do not anticipate any risks in participating in this study. It may be that taking part may cause you to think about your or your families’ wellbeing. If you have any concerns about your health, please seek advice from your GP.

**Do I have to take part?**

No. Participation is completely voluntary, and you can withdraw at any time and without any consequences.

**Will I hear about the results of this project?**

A summary of the survey findings will be placed on the MCRI Genetics of Speech websites. We will also be sharing the results of the survey more widely via academic journals and conferences, and through the development of a briefing to distribute to patient advocacy groups, policy makers, and health providers.

**What will happen to information about me?**

All the information gathered from the surveys will be treated confidentially. Survey data will be held on University of Melbourne computers and retained for 5 years following the publication of our findings.

**Who is funding this project?**

This project is being funded by the Royal Children’s Hospital Foundation grant focused on a centre of Excellence in Speech Genomics. The funding will contribute to survey distribution. The funding agreement ensured researchers’ independence in designing the study, conducting the research, interpreting the data, writing, and publishing the findings.

**Where can I get further information?**

If you would like more information about the project, please contact the researchers;

A/Prof Ilias Goranitis; Email: [ilias.goranitis@unimelb.edu.au](mailto:ilias.goranitis@unimelb.edu.au)

Dr Yan Meng; Email: [yan.meng2@unimelb.edu.au](mailto:yan.meng2@unimelb.edu.au)

You can keep a copy of this form for your records.

**Who can I contact if I have any concerns about the project?**

This research project has been approved by the Human Research Ethics Committee of The University of Melbourne. If you have any concerns or complaints about the conduct of this research project, which you do not wish to discuss with the research team, you should contact the Manager, Human Research Ethics, Research Ethics and Integrity, University of Melbourne, VIC 3010. Tel: +61 3 8344 2073 or Email: HumanEthics-complaints@unimelb.edu.au. All complaints will be treated confidentially. In any correspondence, please provide the name of the research team or the name or ethics ID number of the research project.

**Consent**

I consent to participate in this survey as outlined in the Plain Language Statement ☐

I would like further information about this survey ☐

I do not wish to consent to participate in this survey ☐

**Section 1**

**Please complete the following section to tell us about yourself:**

1. How old are you? _________________ years

1. What is your gender?

Female Male  Prefer to self-describe  Prefer not to say

1. What is your current marital status?

Never married

De facto (living with a partner)

Married

Widowed

Divorced / separated

Other (please specify): _________________

1. What is your highest level of education?

Year 11 or below

Year 12 or equivalent

Certificate

Diploma/advanced diploma

Bachelor’s degree

Graduate diploma/certificate

Post-graduate degree

Other (please specify): _________________

1. What is your household’s annual gross (before tax) income?

Lower than $40,000 per year

$40,000 - $60,000 per year

$60,000 - $80,000 per year

$80,000 - $100,000 per year

$100,000 - $120,000 per year

$120,000 - $140,000 per year

$140,000 - $160,000 per year

Over $160,000 per year

1. Do you have private health insurance?

Yes  No

1. What is your postcode? ______
2. How many children do you have? _______

How many of your children are 15 years or younger? ______

Do any of your children have a speech disorder?

Yes

No

1. Do you have any prior knowledge or experience about severe speech disorders like apraxia or dysarthria?

Yes

No

1. Do you have any prior knowledge or experience about genomic testing?

Yes

No

1. How is your own health today?

Give a number between 0 (worst health you can imagine) to 100 (best health you can imagine): __________

1. How is your child’s health today?

Give a number between 0 (worst health you can imagine) to 100 (best health you can imagine): __________

**Section 2**

Severe speech disorders such as childhood apraxia of speech (CAS), occur in around 1 in 1000 children. CAS is a debilitating condition whereby children require years of intensive speech therapy to learn to speak well enough to be understood. CAS is also associated with longer-term reading and spelling impairments which can affect educational and employment outcomes. Around 1 in 3 children with childhood apraxia of speech have a genetic cause for the condition. Having a child with a severe speech disorder can cause stress for affected children and families, worrying about whether their child will talk, when they will speak normally, or whether they will be understood. Understanding the cause of severe speech disorders can be helpful to avoid a long diagnostic journey of brain imaging or more invasive tests. It can also help in guiding treatment.

**Here is a video of a child with a severe speech disorder (apraxia).**

[insert the patient story video here]

**Section 3**

Genomic testing may have a range of benefits to children and their families, such as providing a diagnosis, informing treatment and family planning, allowing access to clinical trials, and contributing to research, policy and society. However, a genetic diagnosis may also bring challenges, such as concern over the genetic ‘label’, potential disclosure of the results to life insurers, or potential unexpected diagnosis, such as intellectual disability.

This section lists information about seven key characteristics of genomic testing that matter to families who have children with a severe speech disorder. Please read carefully as the next section will ask you to make choices based on how important these are to you.

**Characteristic 1:**

**Number of children who receive genetic diagnosis**

This characteristic tells you how likely it is that a genetic cause for the child’s severe speech disorder will be identified by the test. For example, *“30 out of 100”* means that for every 100 children with similar problems being tested, 30 will receive a genetic diagnosis and 70 will not. Parents find it valuable to understand the cause of their child’s speech disorder because it ends the diagnostic odyssey.

**Please note:**

**Our understanding of genetic conditions is improving all the time. Even when a diagnosis is not made at the time of testing, the genetic data may be reanalysed in future when new analysis methods come to hand and a diagnosis may be made at a later date.**

**Characteristic 2:**

**Knowledge about the child’s future health and development (prognosis)**

This characteristic tells you how much knowledge a genomic result can give you about a child’s future speech and development. For example, for some diagnoses we can provide specific information about how the child’s speech disorder, or their general health, is likely to progress. Sometimes this may be knowledge that the speech disorder will improve or resolve, or alternatively, that the speech disorder will remain despite speech therapy. Other times, the genomic test may reveal that the speech disorder is likely to be accompanied by other health problems or learning difficulties. This knowledge can potentially reduce parents’ uncertainty about the future and enable future planning.

**Please note:**

**The focus of this characteristic is the knowledge gained from genomic information. However, this information may be perceived either positively or negatively regarding the child’s future health development.**

**Characteristic 3:**

**Chance of improving the process of the child’s medical care**

This characteristic tells you how likely the genomic test result will improve the process of the child’s medical care. This characteristic includes guiding the medical management of the child’s speech disorder or other aspects of their general health and development, for example, guiding movement therapies or the intensity of speech therapy, and enabling genetic and prognostic counselling. Other improvements of the process include stopping ineffective medications or referral of the child to a new specialist to monitor specific long-term complications of the condition. These improvements do not directly improve the child’s health but they do enhance the process of their care.

**Please note:**

**Although there might be improvements in the process of the child’s medical care, the extent to which these improvements will change the child’s speech or general health outcomes in the future is uncertain.**

**Characteristic 4:**

**Time between now and when your child does the test**

This characteristic tells you how long you will need to wait to access the test. For parents this time may be a period of uncertainty about the cause of the speech disorder or whether the current therapies are appropriate. Timely access to the test, particularly in young children, may also mean a better opportunity to improve the child’s speech problem or general health.

**Characteristic 5:**

**Cost of testing to you**

Because this is a new test, the Federal Government does not pay for it. This characteristic tells you how much you would need to pay out-of-pocket for the test. The cost could range from a few hundred dollars up to several thousand dollars. The test will be performed once and the payment will be a one-off payment.

**Please note:**

**The survey does not require you to actually pay for the test.**

**To help us accurately value the test, please pay attention to the actual costs of the test presented to you and carefully consider whether this is an amount you would be willing to pay if your doctor recommended a genomic test.**

**Remember that this amount would no longer be available for you to spend on other things, such as grocery or utility bills.**

**Characteristic 6:**

**Allowing access to educational support services**

This characteristic tells you whether the test results will allow the child to access educational support services. The test results may help the child’s school and teachers to better understand their speech disorder and provide tailored support to the child’s educational needs, such as establishing and plan for the educational goals and devising appropriate adaption of curriculum. The test results may also enable early interventions provided in an educational setting, and open choices to the child in special schools.

**Characteristic 7:**

**Enabling access to relevant genetic-based family support or advocacy groups**

This characteristic tells you whether the test results will provide families with the support of genetic-based support or advocacy groups. This characteristic could be sharing knowledge or providing emotional support and social interaction with other families experiencing similar life challenges. For families of children with severe speech disorders and other difficulties, regular meetings and discussion of shared experiences in raising children with the same difficulties is known to improve the wellbeing of both children and their families. These genetic-based groups also help to advocate for children and families with the condition in terms of lobbying for support, recognition and funding. They often support research and development of therapies and knowledge around the condition.

**Section 4**

**In this section, we want to understand how important each of these characteristics is to you.**

**Please imagine that you have a child of 4 years, who is unable to verbally communicate with friends and family. Your doctor has conducted initial investigations and recommends a genomic test to explore if there is an underlying genetic cause for the severe speech disorder.**

We will present eight scenarios. Within each scenario, there will be two different situations: ‘Situation 1’ and ‘Situation 2’. The two situations will differ in seven of the characteristics described earlier.

Please select the situation under which you would prefer your child to have the genomic test. If you would prefer to have the test in both situations, please select the situation where you think genomic testing would be most beneficial. If you would not choose to have the test under either of these situations, please select the option ‘I would not like my child to have a genomic test’.

**Please note:**

**While completing the remainder of the survey, please remember to imagine that you have a child of 4 years, who is unable to verbally communicate with friends and family. Your doctor has conducted initial investigations and recommends a genomic test to explore if there is an underlying genetic cause for the severe speech disorder.**

**An example**

**Under which situation would you like your child to have a genomic test? You can choose either ‘Situation 1’, ‘Situation 2’, or ‘I would not like my child to have a genomic test’**

The person who answered the question below decided that genomic testing would be most beneficial in Situation 1. The decision was made by comparing the characteristics of Situation 1 with those of Situation 2. This person considered that the additional cost involved in Situation 1 was worth the benefit gained from the remaining characteristics.

Please note that in surveys like this, it has been found that some people tend to overestimate or underestimate how much they would really be willing to pay. Thus, they may choose situations that they would not actually prefer in real life. It is important that your choices here are realistic. If you would like to remind yourself during the survey what each of these characteristics mean, point/click on the characteristic of interest and a pop-up box with the description will appear.

|  |  |  |  |  |
| --- | --- | --- | --- | --- |
|  | | Situation 1 | Situation 2 | Neither |
| Number of children who receive genetic diagnosis | | 50 out of 100 | 15 out of 100 |  |
| Knowledge about the child’s future health and development (prognosis) | | Significant knowledge | Limited knowledge |  |
| Chance of improving the process of the child’s medical care | | 60% | 20% |  |
| Time between now and when your child does the test | | 1 month | 3 months |  |
| Cost of testing to you | | A$3,000 | A$500 |  |
| Allowing access to educational support services | | Yes | No |  |
| Enabling access to relevant genetic-based family support groups | | Yes | No |  |
| **Under which situation would you like your child to have a genomic test?** | |  |  |  |
|  |  |  |  |  |

| ***Scenario 1*** | | |  |  |  |
| --- | --- | --- | --- | --- | --- |
|  | | | **Situation 1** | **Situation 2** | **Neither** |
| Number of children who receive genetic diagnosis | | | 30 out of 100 | 30 out of 100 |  |
| Knowledge about the child’s future health and development (prognosis) | | | Moderate knowledge | No knowledge |  |
| Chance of improving the process of the child’s medical care | | | 20% | 40% |  |
| Time between now and when your child does the test | | | 3 months | 3 months |  |
| Cost of testing to you | | | A$4,500 | A$4,500 |  |
| Allowing access to educational support services | | | No | Yes |  |
| Enabling access to relevant genetic-based family support groups | | | Yes | No |  |
| **Under which situation would you like your child to have a genomic test?** | | |  |  |  |
|  | | |  |  |  |
|  | | |  |  |  |
| ***Scenario 2*** | | |  |  |  |
|  | | | **Situation 1** | **Situation 2** | **Neither** |
| Number of children who receive genetic diagnosis | | | 30 out of 100 | 30 out of 100 |  |
| Knowledge about the child’s future health and development (prognosis) | | | No knowledge | Limited knowledge |  |
| Chance of improving the process of the child’s medical care | | | 40% | 40% |  |
| Time between now and when your child does the test | | | 3 months | 3 months |  |
| Cost of testing to you | | | A$500 | A$4,500 |  |
| Allowing access to educational support services | | | Yes | No |  |
| Enabling access to relevant genetic-based family support groups | | | No | Yes |  |
| **Under which situation would you like your child to have a genomic test?** | | |  |  |  |
|  | | |  |  |  |
|  | | |  |  |  |
| ***Scenario 3*** | | |  |  |  |
|  | | | **Situation 1** | **Situation 2** | **Neither** |
| Number of children who receive genetic diagnosis | | | 50 out of 100 | 15 out of 100 |  |
| Knowledge about the child’s future health and development (prognosis) | | | Limited knowledge | Significant knowledge |  |
| Chance of improving the process of the child’s medical care | | | 60% | 20% |  |
| Time between now and when your child does the test | | | 6 months | 1 month |  |
| Cost of testing to you | | | A$3,000 | A$500 |  |
| Allowing access to educational support services | | | No | Yes |  |
| Enabling access to relevant genetic-based family support groups | | | No | Yes |  |
| **Under which situation would you like your child to have a genomic test?** | | |  |  |  |
|  | | |  |  |  |
| ***Scenario 4*** | | |  |  |  |
|  | | | **Situation 1** | **Situation 2** | **Neither** |
| Number of children who receive genetic diagnosis | | | 15 out of 100 | 50 out of 100 |  |
| Knowledge about the child’s future health and development (prognosis) | | | Significant knowledge | No knowledge |  |
| Chance of improving the process of the child’s medical care | | | 40% | 40% |  |
| Time between now and when your child does the test | | | 1 month | 6 months |  |
| Cost of testing to you | | | A$500 | A$500 |  |
| Allowing access to educational support services | | | No | Yes |  |
| Enabling access to relevant genetic-based family support groups | | | No | Yes |  |
| **Under which situation would you like your child to have a genomic test?** | | |  |  |  |
|  | | |  |  |  |
|  | | |  |  |  |
| ***Scenario 5*** | | |  |  |  |
|  | | | **Situation 1** | **Situation 2** | **Neither** |
| Number of children who receive genetic diagnosis | | | 50 out of 100 | 15 out of 100 |  |
| Knowledge about the child’s future health and development (prognosis) | | | Moderate knowledge | Limited knowledge |  |
| Chance of improving the process of the child’s medical care | | | 60% | 40% |  |
| Time between now and when your child does the test | | | 1 month | 6 months |  |
| Cost of testing to you | | | A$3,000 | A$1,500 |  |
| Allowing access to educational support services | | | No | Yes |  |
| Enabling access to relevant genetic-based family support groups | | | No | Yes |  |
| **Under which situation would you like your child to have a genomic test?** | | |  |  |  |
|  | | |  |  |  |
|  | | |  |  |  |
| ***Scenario 6*** | | |  |  |  |
|  | | | **Situation 1** | **Situation 2** | **Neither** |
| Number of children who receive genetic diagnosis | | | 30 out of 100 | 30 out of 100 |  |
| Knowledge about the child’s future health and development (prognosis) | | | Significant knowledge | No knowledge |  |
| Chance of improving the process of the child’s medical care | | | 20% | 20% |  |
| Time between now and when your child does the test | | | 3 months | 3 months |  |
| Cost of testing to you | | | A$4,500 | A$4,500 |  |
| Allowing access to educational support services | | | Yes | No |  |
| Enabling access to relevant genetic-based family support groups | | | No | Yes |  |
| **Under which situation would you like your child to have a genomic test?** | | |  |  |  |
|  | | |  |  |  |
|  | | |  |  |  |
| ***Scenario 7*** | | |  |  |  |
|  | | | **Situation 1** | **Situation 2** | **Neither** |
| Number of children who receive genetic diagnosis | | | 15 out of 100 | 50 out of 100 |  |
| Knowledge about the child’s future health and development (prognosis) | | | Limited knowledge | Significant knowledge |  |
| Chance of improving the process of the child’s medical care | | | 20% | 60% |  |
| Time between now and when your child does the test | | | 1 month | 6 months |  |
| Cost of testing to you | | | A$500 | A$1,500 |  |
| Allowing access to educational support services | | | No | Yes |  |
| Enabling access to relevant genetic-based family support groups | | | Yes | No |  |
| **Under which situation would you like your child to have a genomic test?** | | |  |  |  |
|  | | |  |  |  |
|  | | |  |  |  |
| ***Scenario 8*** | | |  |  |  |
|  | | | **Situation 1** | **Situation 2** | **Neither** |
| Number of children who receive genetic diagnosis | | | 30 out of 100 | 30 out of 100 |  |
| Knowledge about the child’s future health and development (prognosis) | | | Significant knowledge | Limited knowledge |  |
| Chance of improving the process of the child’s medical care | | | 40% | 20% |  |
| Time between now and when your child does the test | | | 3 months | 3 months |  |
| Cost of testing to you | | | A$4,500 | A$4,500 |  |
| Allowing access to educational support services | | | No | Yes |  |
| Enabling access to relevant genetic-based family support groups | | | No | Yes |  |
| **Under which situation would you like your child to have a genomic test?** | | |  |  |  |
|  | | |  |  |  |
|  | | |  |  |  |
| ***Scenario 9*** | | |  |  |  |
|  | | | **Situation 1** | **Situation 2** | **Neither** |
| Number of children who receive genetic diagnosis | | | 30 out of 100 | 30 out of 100 |  |
| Knowledge about the child’s future health and development (prognosis) | | | No knowledge | Limited knowledge |  |
| Chance of improving the process of the child’s medical care | | | 40% | 60% |  |
| Time between now and when your child does the test | | | 3 months | 3 months |  |
| Cost of testing to you | | | A$3,000 | A$3,000 |  |
| Allowing access to educational support services | | | Yes | No |  |
| Enabling access to relevant genetic-based family support groups | | | No | Yes |  |
| **Under which situation would you like your child to have a genomic test?** | | |  |  |  |
|  | | |  |  |  |
|  | | |  |  |  |
| ***Scenario 10*** | | |  |  |  |
|  | | | **Situation 1** | **Situation 2** | **Neither** |
| Number of children who receive genetic diagnosis | | | 15 out of 100 | 50 out of 100 |  |
| Knowledge about the child’s future health and development (prognosis) | | | No knowledge | Moderate knowledge |  |
| Chance of improving the process of the child’s medical care | | | 60% | 40% |  |
| Time between now and when your child does the test | | | 6 months | 1 month |  |
| Cost of testing to you | | | A$1,500 | A$1,500 |  |
| Allowing access to educational support services | | | Yes | No |  |
| Enabling access to relevant genetic-based family support groups | | | Yes | No |  |
| **Under which situation would you like your child to have a genomic test?** | | |  |  |  |
|  | | |  |  |  |
|  |  |  |  |  |  |
| ***Scenario 11*** | | |  |  |  |
|  | | | **Situation 1** | **Situation 2** | **Neither** |
| Number of children who receive genetic diagnosis | | | 50 out of 100 | 15 out of 100 |  |
| Knowledge about the child’s future health and development (prognosis) | | | Limited knowledge | Significant knowledge |  |
| Chance of improving the process of the child’s medical care | | | 40% | 60% |  |
| Time between now and when your child does the test | | | 1 month | 6 months |  |
| Cost of testing to you | | | A$3,000 | A$1,500 |  |
| Allowing access to educational support services | | | Yes | No |  |
| Enabling access to relevant genetic-based family support groups | | | Yes | No |  |
| **Under which situation would you like your child to have a genomic test?** | | |  |  |  |
|  | | |  |  |  |
|  |  |  |  |  |  |
| ***Scenario 12*** | | |  |  |  |
|  | | | **Situation 1** | **Situation 2** | **Neither** |
| Number of children who receive genetic diagnosis | | | 50 out of 100 | 15 out of 100 |  |
| Knowledge about the child’s future health and development (prognosis) | | | Limited knowledge | No knowledge |  |
| Chance of improving the process of the child’s medical care | | | 20% | 60% |  |
| Time between now and when your child does the test | | | 6 months | 1 month |  |
| Cost of testing to you | | | A$500 | A$1,500 |  |
| Allowing access to educational support services | | | Yes | No |  |
| Enabling access to relevant genetic-based family support groups | | | Yes | No |  |
| **Under which situation would you like your child to have a genomic test?** | | |  |  |  |

**Feedback**

How did you find the questions in this survey?

☐ Easy ☐ Moderate ☐ Difficult

Have you got any comments about this questionnaire that you would like to share with us?

**Acknowledgement**

Thank you for taking the time to complete our survey.

1. Eising, Else, Amaia Carrion-Castillo, Arianna Vino, Edythe A. Strand, Kathy J. Jakielski, Thomas S. Scerri, Michael S. Hildebrand et al. "A set of regulatory genes co-expressed in embryonic human brain is implicated in disrupted speech development." Molecular psychiatry 24, no. 7 (2019): 1065-1078. [↑](#footnote-ref-1)
